# Study on the Low-Temperature Performance Evaluation Indicators of Asphalt Binder Based on the Poker Chip Test

**DOI:** 10.3390/ma18061322

**Published:** 2025-03-17

**Authors:** Meng Guo, Chenlu Sun, Yiqiao Wan, Xiuli Du

**Affiliations:** 1 State Key Laboratory of Bridge Safety and Resilience, Beijing University of Technology, Beijing 100124, China; sunchenlu0910@emails.bjut.edu.cn (C.S.); duxiuli@bjut.edu.cn (X.D.); 2 The Key Laboratory of Urban Security and Disaster Engineering of Ministry of Education, Beijing University of Technology, Beijing 100124, China; 3 Department of Informatics, Beijing University of Technology, Beijing 100124, China; yiqiao1009@emails.bjut.edu.cn

**Keywords:** the poker chip test, low-temperature cracking resistance, tensile strength, ultimate tensile strain

## Abstract

Low-temperature cracking is a primary failure mode of asphalt pavement. The poker chip test provides a straightforward and efficient approach to simulating the film state of asphalt binders in asphalt structures. By measuring the tensile strength and ultimate tensile strain of the binder film, this test can effectively evaluate the cracking resistance and ductility of asphalt binders. Accordingly, this study employed the poker chip test to analyze the evolutions of low-temperature cracking resistance under various aging levels. To ensure the reliability of tensile strength and ultimate tensile strain, a Pearson correlation analysis was conducted between the two indicators and the traditional low-temperature performance evaluation indicators: stiffness modulus, creep rate, and the Glover-Rowe (G-R) parameter. The results indicate that the tensile strength and ultimate tensile strain of styrene–butadiene–styrene (SBS)-modified asphalt are higher than those of 70# base asphalt at the same aging level. With increasing aging time, the tensile strength of both SBS-modified asphalt and 70# base asphalt increases, while the ultimate tensile strain decreases. Additionally, the tensile strength and ultimate tensile strain are sensitive to changes in asphalt binder types and aging levels. They have a good linear correlation with stiffness modulus and creep rate, with correlation coefficients exceeding 0.9. Due to the distinct characteristics represented, the correlation between the two indicators and the G-R parameter is relatively weaker, with correlation coefficients exceeding 0.7. The findings of this study demonstrate that tensile strength and ultimate tensile strain are effective indicators for assessing the low-temperature performance of asphalt binders. They can serve as substitute indicators of stiffness modulus and creep rate, respectively.

## 1. Introduction

Asphalt pavement is widely used in modern highway construction. However, diseases such as ruts, cracks, and water damage may frequently occur throughout the service life of asphalt pavement. Among the key factors affecting asphalt pavement performance, low temperature and aging play critical roles. These two factors increase the stiffness and brittleness of asphalt binders, resulting in a significant reduction in the cracking resistance [1,2].

Asphalt binders play the role of binding mineral aggregate in asphalt mixtures. The deterioration of asphalt binder low-temperature cracking resistance will significantly shorten the service life of asphalt pavement [3,4,5]. Therefore, it is necessary to evaluate the cracking resistance of asphalt binders under different aging levels more accurately through appropriate evaluation methods and indicators.

The penetration-based standard is the earliest low-temperature performance evaluation system in the world [6]. The penetration index reflects the temperature sensitivity and stiffness of asphalt binder. The Finnish Asphalt Pavement Research Program (ASTO) used the penetration index at −5 °C as a technical parameter to evaluate the low-temperature cracking resistance of asphalt binders [7]. However, asphalt binders become brittle and hard at low temperatures, making the measurement more difficult and increasing the measurement errors. As a result, the method fails to differentiate the types of asphalt binders clearly. Furthermore, although the penetration test is simple to conduct, it cannot accurately reflect the viscoelastic properties of asphalt binders.

Ductility is another important indicator for assessing the low-temperature performance of asphalt binders. Asphalt binders with greater ductility exhibit better low-temperature performance [8]. Many countries established ductility standards at 0 °C, 4 °C, 5 °C, 7 °C, 10 °C, etc. However, at higher test temperatures, asphalt binders exhibit higher viscosity, failing to reflect their brittle fracture characteristics at low temperatures. Conversely, at lower test temperatures, the ductility differences between various asphalt binders diminish, making it challenging to distinguish between the types of asphalt binders. Therefore, despite its simplicity and quick execution, the ductility test requires careful selection of parameters such as test temperature and tensile rate to ensure accurate results [9]. For modified asphalt, ductility is influenced by various factors, including the base asphalt binders and the type and content of the modifier. Studies indicated that the correlation between ductility and low-temperature performance of modified asphalt was relatively weak [10,11,12,13]. To address this limitation, researchers proposed the force–ductility test as an extension of the traditional ductility test [14]. This method was considered more suitable for evaluating the low-temperature performance of modified asphalt, offering more comprehensive and diverse evaluation indicators [15]. However, due to the imperfect test methods, strict test conditions, and complex data processing, it was not widely adopted.

Some studies suggested that the Fraass breaking point could reflect the low-temperature brittleness of asphalt binders [16,17]. A lower breaking point typically indicates better low-temperature performance. However, when the asphaltene content is low and the wax content is high, even if the breaking point meets the required standard, asphalt pavement may still experience severe cracking [18]. Not only that, the breaking point test exhibits poor repeatability, as its results are influenced by various factors, including testing equipment, cooling conditions, and asphalt binder aging levels [19]. To improve this methodology, some scholars proposed using the equivalent breaking point as an alternative indicator for evaluating the low-temperature cracking resistance of asphalt binders [18]. However, this method is significantly affected by the choice of regression equations [20]. Additionally, it cannot effectively assess the low-temperature performance of modified asphalt. Therefore, the equivalent breaking point still has some limitations.

Some other studies indicated that low-temperature stiffness was a critical indicator for evaluating the low-temperature cracking resistance of asphalt mixtures. The Strategic Highway Research Program (SHRP) proposed the bending beam rheometer (BBR) test using the stiffness modulus (*S*) and creep rate (*m*) as evaluation parameters for the low-temperature performance of asphalt binders [21]. However, the BBR test cannot accurately simulate the stress state of asphalt mixtures under low-temperature conditions [22,23]. The direct tensile test (DTT) was proposed as a supplement to the BBR test by SHAP [24]. However, there are still some problems with the DTT test, such as high requirements for sample preparation and difficulties in measuring small displacements.

In reality, asphalt binders exist as thin films in asphalt mixtures.

The thickness and condition of these films are related to the characteristics and quantity of the mineral fillers, as well as the interactions between the fillers and the asphalt binders [25]. When asphalt mixtures undergo aging, the asphalt films also age [26]. Research shows that the effective penetration depth of thermal oxidation for the mixtures can reach 1 mm [27]. Therefore, it is necessary to study the performance of asphalt binders in the film state. However, current evaluation methods fail to fully simulate the actual state of the binder film in asphalt pavement. Therefore, Gent and Lindley et al. [28] first conducted experiments on a thin film of soft elastic material between rigid substrates, investigating the stress state of the material during the stretching process. This configuration is commonly referred to as the poker chip geometry. The results showed that the hydrostatic tensile stress during the stretching process was the primary cause of failure, and the tensile stress was closely related to the elastic modulus of the material. Lindley [29] further extended their work and discovered that the failure strain measured in the poker chip test was 10 times smaller than that obtained from a uniaxial tensile test, and the tensile stress was two to three times higher. These findings indicate that the mechanical characteristics of asphalt binders in the film state differ significantly from those in the trabecular state. Consequently, the poker chip test provides a more accurate simulation of the tensile damage experienced by asphalt binders on the road.

The tensile strength, stress relaxation capacity, and ductility of asphalt binder are closely related to the low-temperature cracking resistance. Some researchers suggested that the poker chip test could effectively simulate the stress state of asphalt binders in asphalt mixtures and allow for the direct measurement of tensile strength [30]. Other studies demonstrated that the failure of asphalt binder film was typically cohesive rather than adhesive [31]. Moreover, studies showed that the low-temperature cracking resistance of asphalt pavement largely depended on the low-temperature performance of the asphalt binder itself [32,33]. Sultana et al. [30] conducted the poker chip test by changing the content of four components in asphalt binders and observed that the failure characteristics of the film were influenced by the asphalt composition. Their findings showed that higher polar component content in asphalt resulted in greater tensile strength of the film, along with smaller and more numerous cavities in the failure section. In addition, Angelo et al. [34] confirmed that the poker chip test exhibited good repeatability, and its test results were sensitive to factors such as aging time and modifier.

In summary, current evaluation methods of asphalt binder low-temperature cracking resistance still have some limitations, including complex procedures, intricate sample preparation, high technical requirements, and limited universality. It is necessary to put forward simple, applicable, and accurate indicators to evaluate the low-temperature performance of asphalt binders. The poker chip test can effectively simulate the stress state of asphalt binders, and parameters such as tensile strength and ultimate tensile strain can evaluate the crack resistance and ductility of asphalt binders. This study used the poker chip test to analyze failure cross sections, tensile strength, and ductility under different aging levels. A Pearson correlation analysis was also conducted to compare the indicators from the poker chip test and frequency sweep tests to verify the poker chip test’s feasibility for low-temperature cracking resistance evaluation.

## 2. Materials and Methods

### 2.1. Raw Materials

In this study, 70# base asphalt and SBS-modified asphalt (prepared by blending the same 70# base asphalt with 4.5% linear SBS modifier through high-speed shearing at 170 °C for 2 h) were used.

The SBS modifier reinforces asphalt binders by establishing a continuous elastomeric network through physical crosslinking, thereby achieving a synergistic enhancement in both ductility and crack resistance. This mechanism allows the material to alleviate stress concentration through viscoelastic recovery while providing critical cohesive strength to withstand low-temperature cracking.

The basic properties of the above asphalt binders were tested according to the American Society for Testing and Materials. The specific test results are shown in Table 1 and Table 2.

### 2.2. Testing Methods

#### 2.2.1. Aging Test

This paper used indoor aging tests to simulate the aging behavior of asphalt binders in the process of production, transportation, paving, and service. Short-term aging tests and long-term aging tests were conducted on the two asphalt binders according to ASTM D2872 and ASTM D6521.

The rolling thin film oven test (RTFOT) was used to simulate the aging behavior of asphalt binders in the process of production, transportation, and paving. The test rate was set to 15 ± 0.2 r/min, the temperature was set to 163 ± 0.5 °C, and the time was set to 75 min.

The Prentex 9300 pressure aging vessel was used to simulate the thermal oxygen aging of asphalt binder. The air pressure in the aging vessel was set to 2.1 ± 0.1 MPa, the aging temperature was set to 100 °C, and the aging time was set to 10 h, 20 h, and 40 h. In this study, “UA” was used to represent unaged asphalt binder, and “PAV 0 h”, “PAV 10 h”, “PAV 20 h”, and “PAV 40 h” represented short-term aging, long-term aging 10 h, long-term aging 20 h, and long-term aging 40 h, respectively.

#### 2.2.2. The Poker Chip Test

**Figure 1:**
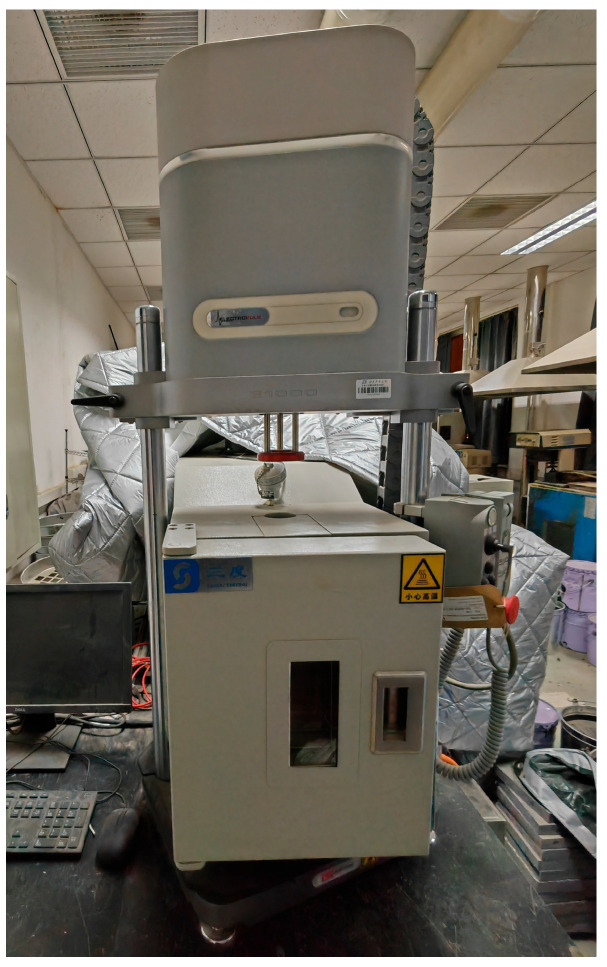
The testing Instrument E1000.

The tensile strength and ductility of asphalt binder in a thin film state were tested by the poker chip test. In the test, the asphalt binder film was placed between two parallel rigid metal plates and subjected to uniaxial tension. The test was conducted using the E1000 loading device, an all-electric instrument designed for dynamic and static testing, manufactured by Instron (Norwood, MA, USA). This instrument was capable of providing an axial dynamic load with a maximum of 1 kN. Figure 1 shows the E1000 used in the poker chip test.

The specimen preparation method adopted in this study referred to the operation method proposed by Angelo et al. [34]. The bottom plate had a flat face of 58 mm diameter with a 4 mm deep lip along the circumference to retain liquid binder within it. The top plate had a flat face of 50 mm diameter. Firstly, the stainless-steel plates, alignment pins, and spacer dowels were heated in the oven at 165 °C for not less than 45 min. Secondly, 4.5 ± 0.5 g asphalt binder was placed in the heated bottom plate and put back in the oven. After being heated at 165 °C for 15 min, the binder melted and spread over the whole disk surface of the bottom plate to form a film. Then three spacer dowels with a diameter of 1 mm were placed at a position of 1/2 radius in the bitumen film at an interval of 120°. The bottom plate was put back into the oven and kept at 165 °C for another 5 min. Finally, the top plate was covered by the bottom plate containing the asphalt film. The whole specimen was put back in the oven and held at 165 °C for 5 min. At this time, due to the gravity action of the top plate and the positioning action of the spacer dowels, the thickness of the asphalt film was controlled to 1 mm.

According to the test results of Sharmin Sultana et al., the poker chip test mechanism could make the asphalt film sandwiched between plates in a state of triaxial stress. The stress field was related to the diameter-to-thickness ratio of the film and Poisson’s ratio of asphalt binder. When the film diameter to thickness ratio exceeded 40, the asphalt film was in a uniform triaxial stress state, and the critical tensile stress was related to its properties but not affected by the film thickness and other factors [30]. The diameter-to-thickness ratio of the binder film used in this paper was 50, ensuring that the asphalt film was in a uniform triaxial stress state. The prepared specimens needed to be cooled to 25 °C and kept at 25 °C for at least 2 h.

**Figure 2:**
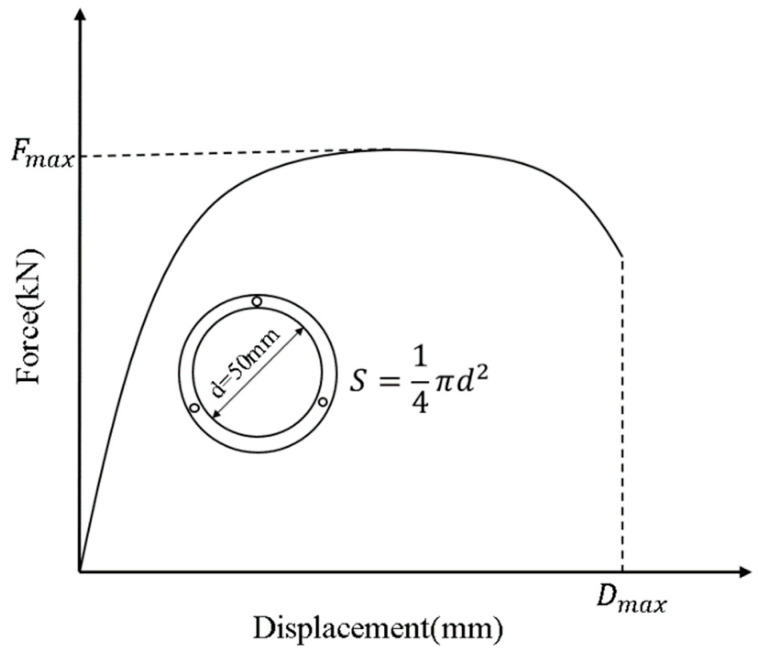
Schematic diagram of the force–displacement curve and plate parameters.

According to the results of the poker chip test, a force–displacement curve could be plotted. In addition, the tensile strength and ductility of asphalt binder films can be evaluated according to the force–displacement curve. Figure 2 is the schematic diagram of the force–displacement curve and plate parameters.

As shown in Figure 2, the maximum tensile force is denoted as $F_{max}$ (kN), the maximum tensile displacement as $D_{max}$ (mm), and the stretching cross section as a standard circle with a diameter *d* (mm). In this study, the stretching cross-sectional diameter is 50 mm, and the cross-sectional area *S* (mm^2^) can be calculated accordingly. The tensile strength T and ultimate tensile strain $\sigma_{m}$ are used to evaluate the cracking resistance and deformation capacity of asphalt binders at low temperatures. Tensile strength is defined as the ratio of the maximum tensile force to the stretching cross-sectional area, reflecting the ability to resist low-temperature cracking. Since the initial displacement has been set to zero before stretching, tensile displacement is equivalent to tensile strain. The ultimate tensile strain, corresponding to the end of the cracking stage, characterizes the deformation capacity of the binder. The calculation methods are provided in Equations (1) and (2).

(1) $$T=\frac{F_{max}}{S}\times 10^{3}$$

(2) $$\sigma_{m}=D_{max}\times 100\%$$

where T is the tensile strength of asphalt film (MPa), $F_{max}$ is the maximum tensile force (kN), S is the stretching cross-sectional area (mm^2^), $\sigma_{m}$ is the ultimate tensile strain (%), and $D_{max}$ is the tensile strain corresponding to the end of the cracking stage (mm).

#### 2.2.3. Frequency Sweep Test

**Figure 3:**
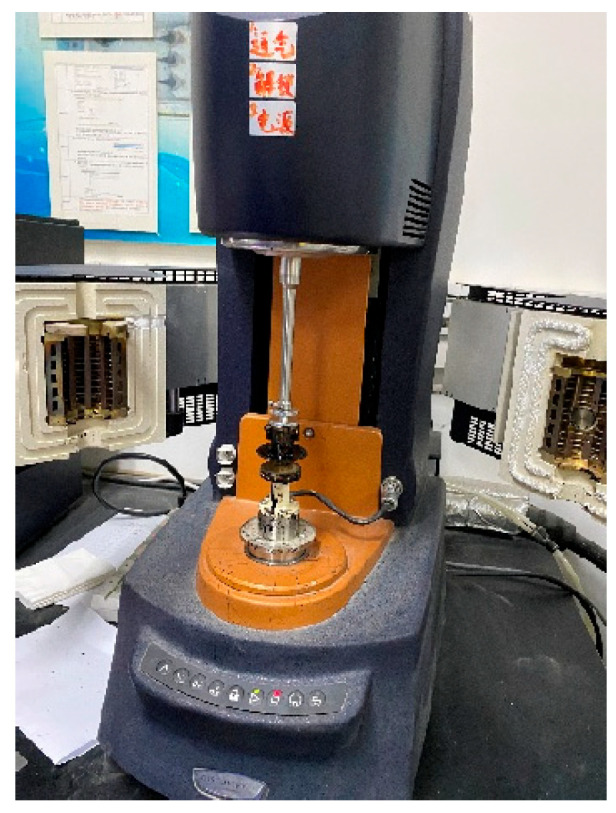
The DHR-2 dynamic shear rheometer.

In this study, the Discovery Series DHR-2 dynamic shear rheometer, manufactured by TA Instruments (New Castle, DE, USA), was used to conduct frequency sweep tests at medium and low temperatures to evaluate the cracking resistance of aging asphalt binders. Figure 3 shows the dynamic shear rheometer with a sample.

Studies have demonstrated that the G-R parameter correlates well with the low-temperature performance of asphalt mixture and field cracking [35,36]. Furthermore, it effectively reflects the impact of thermal-oxidative aging on asphalt binder cracking resistance [37]. In this study, 8 mm parallel plates were used for the frequency sweep test in the medium temperature range. The complex modulus and phase angle master curves at 15 °C were constructed according to the time–temperature equivalent principle to calculate the G-R parameter at 15 °C and 0.005 rad/s of different aging times. The calculation Equations (3) and (4) are as follows:

(3) $$Glover\;parameter=\frac{G\prime }{\left(\eta^{\prime }/G\prime \right)}$$

where G′ and $\eta^{\prime }$ are the storage modulus (kPa) and dynamic viscosity (Pa·s) at 15 °C and 0.005 rad/s, respectively.

(4) $$G-R\;parameter=\frac{{G^{*}\times (\cos\delta )}^{2}}{\sin\delta }$$

where $G^{*}$ and δ are the complex modulus (kPa) and phase Angle (°) at 15 °C and 0.005 rad/s, respectively.

The parameters of the medium temperature frequency sweep test were set as follows:

Controlled strain: 1%;

Scanning frequency: 100 rad/s~0.1 rad/s;

Temperatures: 5 °C, 15 °C, 25 °C;

Gap and diameter: 2 mm and 8 mm.

To address the challenges of sample preparation and temperature control in the BBR test, the Western Research Institute (WRI) proposed to use a 4 mm-parallel-plate frequency sweep test instead of the BBR test to measure the low-temperature stiffness modulus and creep rate of asphalt binders. To ensure consistency with traditional BBR evaluation indicators, various conversion methods were developed to calculate equivalent stiffness modulus and creep rate from the frequency sweep test results [38,39,40]. Experiments have shown that the transformation method proposed by Ninomiya and Ferry exhibits a strong linear correlation with the BBR test results [40,41,42]. The main conversion steps include transforming frequency domain responses into time domain responses and converting shear responses into uniaxial responses. The process begins by fitting the complex modulus master curve using Equation (3) at the test temperature. Secondly, Equation (4) is applied to convert complex modulus $G^{*}(\omega )$ into complex compliance $J^{*}\left(\omega \right)$, and frequency domain data is transformed into time domain data via Equation (5). Finally, Equation (6) is used to convert shear compliance $J\left(t\right)$ into tensile compliance $D\left(t\right)$, where v, the Poisson’s ratio of asphalt, is set to 0.35. The equivalent stiffness modulus $S\left(t\right)$ for the BBR test is obtained by the reciprocal of tensile compliance, and the calculation of the creep rate m follows the same method as in the BBR test.

(5) $$\log\left|G^{*}(\omega )\right|=\eta +\frac{\alpha }{1+e^{\beta +\gamma \log\left(\omega \right)}}$$

(6) $$J^{*}\left(\omega \right)=\frac{1}{G^{*}(\omega )}$$

(7) $$J\left(t\right)=J^{\prime }\left(\omega \right)+0.4J^{\prime \prime }\left(0.4\omega \right)-0.014J^{\prime \prime }\left(10\omega \right)$$

(8) $$D\left(t\right)=\frac{J(t)}{2(1+v)}$$

(9) $$S\left(t\right)=\frac{1}{D(t)}$$

In Equations (5)–(9), ω is the loading frequency at the reference temperature, η is the minimum value of $G^{*}$, η+α is the maximum value of $G^{*}$, β and γ are the coefficients, t is the loading time, $J^{\prime }\left(\omega \right)$ is the storage compliance, and $J^{\prime \prime }\left(\omega \right)$ is the loss compliance.

According to the equivalent conversion method, a low-temperature frequency sweep test was used to test the cracking resistance of asphalt binder. The test parameters were set as follows:

Controlled strain: 0.1%;

Scanning frequency: 100 rad/s~0.2 rad/s;

Temperatures: −6 °C, −12 °C, −18 °C;

Gap and diameter: 2.2 mm and 4 mm.

## 3. Results and Discussion

### 3.1. Analysis of the Poker Chip Test Results

#### 3.1.1. Failure Cross Sections

**Figure 4:**
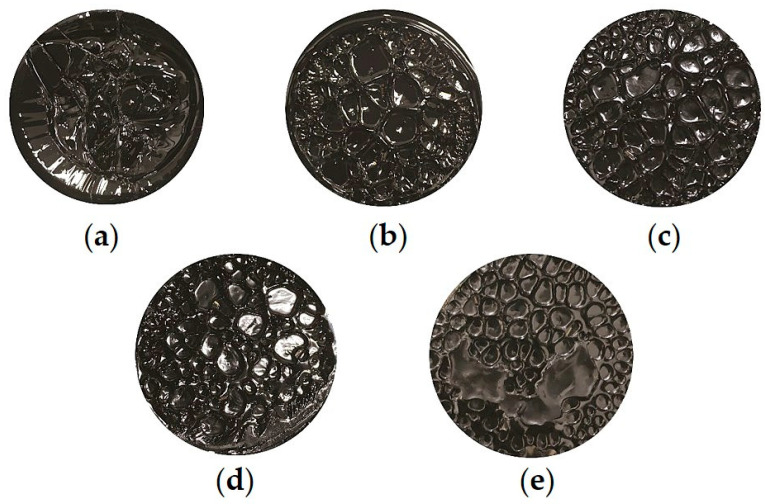
Failure cross sections of 70# base asphalt: (**a**) Unaged; (**b**) Short-term aged; (**c**) 10 h long-term aged; (**d**) 20 h long-term aged; (**e**) 40 h long-term aged.

**Figure 5:**
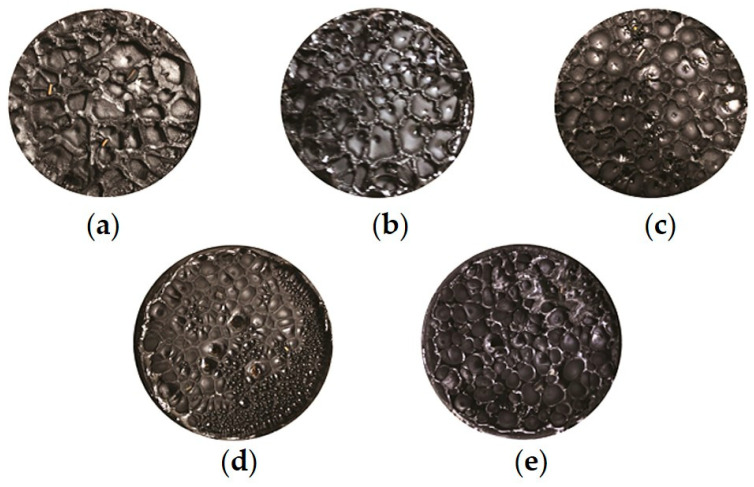
Failure cross sections of SBS-modified asphalt: (**a**) Unaged; (**b**) Short-term aged; (**c**) 10 h long-term aged; (**d**) 20 h long-term aged; (**e**) 40 h long-term aged.

Figure 4 and Figure 5 show the failure cross sections of 70# base asphalt and SBS-modified asphalt films after tensile fracture, respectively.

It can be observed from Figure 4 and Figure 5 that, except for the cross section of unaged 70# base asphalt, cavities appear on the failure cross sections of all the other asphalt binders, and the size and number of these cavities vary depending on the aging levels.

**Figure 6:**
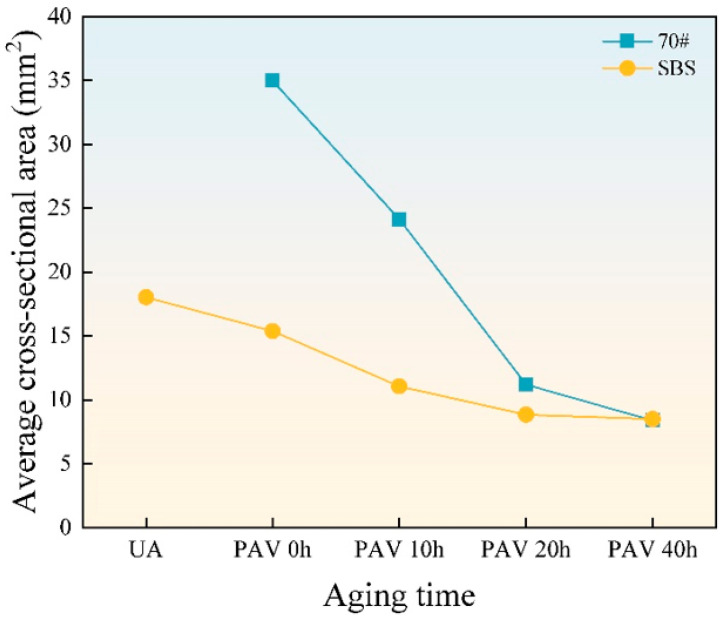
The average cross-sectional area of the cavities.

Figure 6 presents the line diagram of the average cross-sectional area of cavities, calculated using ImageJ 1.52a. The symbols in the upper right corner of the figure represent the asphalt binder types, where “70#” represents 70# base asphalt and “SBS” represents SBS-modified asphalt. The designations below the horizontal axis represent the aging degrees of asphalt binders. “UA” represents unaged asphalt binder. “PAV 0 h”, “PAV 10 h”, “PAV 20 h”, and “PAV 40 h” represents short-term aging, long-term aging 10 h, long-term aging 20 h, and long-term aging 40 h, respectively.

For unaged 70# base asphalt, the binder at the edge of the cross section flows to the center due to its good fluidity, preventing the formation of cavities with clear boundaries. As a result, the average cross-sectional area could not be measured. As shown in Figure 1, Figure 2 and Figure 3, the number of cavities on the failure cross sections of both asphalt binders increases with aging time, while the average cross-sectional area decreases. Compared to SBS-modified asphalt, 70# base asphalt exhibits more significant changes in cavity size.

The formation and growth of cavities are the main factors of film failure. When the loading mode is changed to load control, axial tension increases until it exceeds the maximum tension the asphalt film can withstand. When the material at stress concentration points fails, cavities initially form. Filaments or thin films develop between the top and bottom plates, causing actual stress to rise. Cavities then expand until their boundaries connect, ultimately resulting in the filament or film being pulled apart. The increase in polar molecules, particularly asphaltenes, increases stiffness and reduces ductility, thereby lowering the tensile strain the asphalt binder film can endure [43,44,45]. Apart from that, the aggregation tendency of asphaltene molecules leads to an uneven distribution of components within the binder film [46,47]. This unevenness increases stress concentration areas, promoting the formation of more numerous and denser cavities, and causing the asphalt film to fail at lower strain levels.

The failure cross section of unaged 70# base asphalt shows no obvious cavities due to high fluidity. After short-term aging, cavities of 70# base asphalt are mostly concentrated at the center of the cross section. As aging time increases, the cavities expand across the entire section. This occurs because aging reduces the content of light components (saturates and aromatics), increases the elastic ratio of asphalt binder, and decreases its fluidity. Under increasing axial tension, the ability of the aged asphalt binder at the edges to resist flowing toward the center improves, causing cavities to spread across the entire cross section after long-term aging.

Cavities of SBS-modified asphalt are relatively evenly distributed across the entire failure cross section. Under the same aging time, the cavity boundaries of SBS-modified asphalt are clearer, and the failure cross sections are flatter. As a thermoplastic elastomer, SBS absorbs light components and swells during high-temperature shearing and mixing with base asphalt. This process increases the elastic proportion, improving the mechanical properties of asphalt binder. Furthermore, SBS increases the number of crosslinking structures and intermolecular forces in the modified asphalt, resulting in higher cohesion of modified asphalt. Compared to 70# base asphalt, SBS-modified asphalt has lower fluidity at the same temperature. During axial stretching, the flow of SBS-modified asphalt from the edges toward the center is less evident. Therefore, the failure cross sections of SBS-modified asphalt are flatter and the boundaries are clearer.

#### 3.1.2. Tensile Strength and Ultimate Tensile Strain

**Figure 7:**
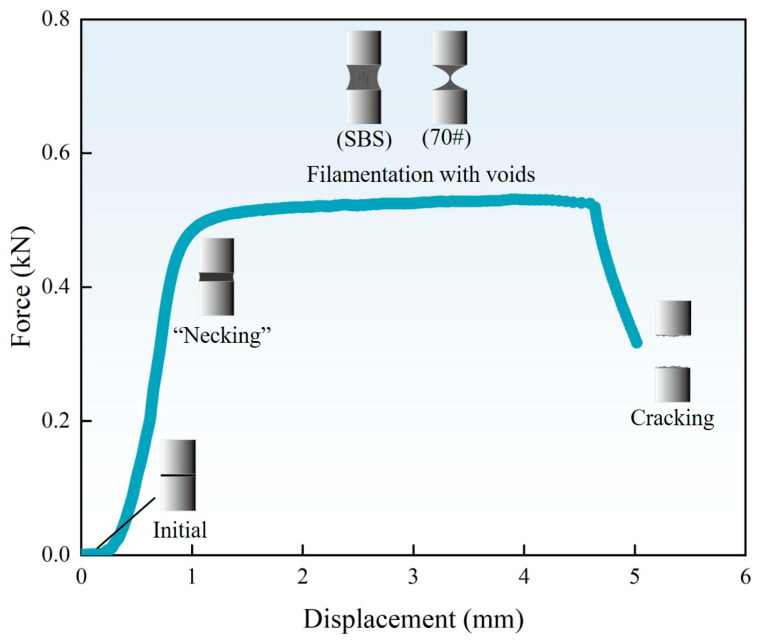
Diagram of the force–displacement curve and the stretching process.

During the pulling process, E1000 recorded the displacement and axial force. Figure 7 is a typical force–displacement diagram curve of the poker chip test measured by E1000.

As shown in Figure 7, the stretching process can be divided into four stages. The first is the initial stage. The asphalt binder film exhibits no significant changes under displacement-controlled loading. Both the force and displacement have a very small range of variations. As the process progresses to the elastic stage, the axial tensile force increases, causing the binder film to undergo slight necking. It is worth noting that the necking represents the asphalt film morphology rather than the necking stage in the stress–strain curve. The cross-sectional area gradually decreases from the edges toward the center, and the force and displacement increase proportionally, producing a nearly linear force–displacement curve with a steep slope. When the displacement increases slightly, the force rapidly reaches the tensile limit of the film and enters the “filamentation with voids” stage. In this stage, the tensile force stabilizes while displacement continues to increase, creating a “platform region” in the force–displacement curve. The binder film undergoes significant necking, and cavities form at the cross sections near the top and bottom plates. Meanwhile, a thin wall forms in the middle of the stretched film. For unaged and short-term aged base asphalt, the low elastic ratio and high fluidity result in the formation of filaments. As displacement increases further, cavities expand, voids develop on the thin wall, and the stretching section shrinks, leading to a rise in actual stress. Finally, the process enters the cracking stage, where the voids on the thin wall connect, and cavity boundaries at the top and bottom plates merge. The thin wall gradually transitions into filaments, which eventually break. The tensile force then decreases rapidly to zero, marking the complete failure of the binder film.

**Figure 8:**
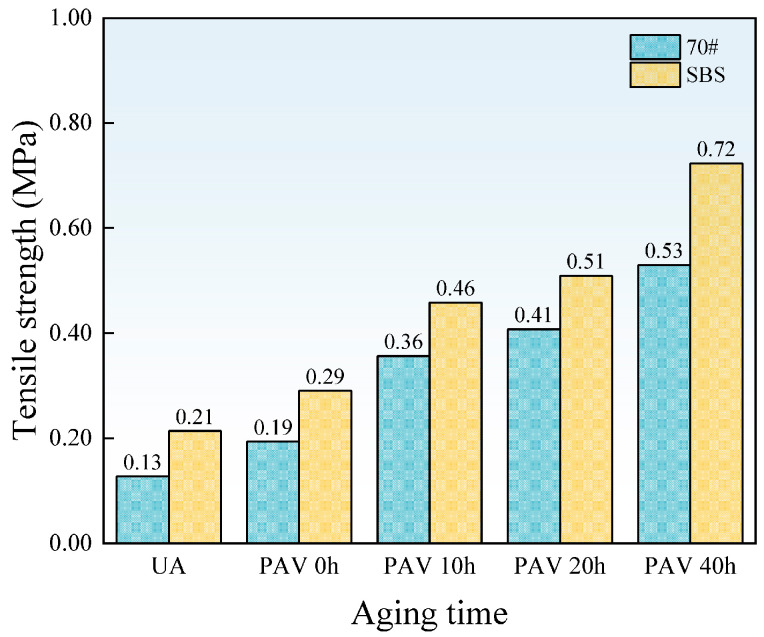
The tensile strength of asphalt films.

Figure 8 shows the evolutions of the tensile strength under different aging times.

As shown in Figure 8, the tensile strength of both 70# base asphalt and SBS-modified asphalt increases with aging time. This is because aging increases the hardness and brittleness of the asphalt binder, resulting in higher tensile strength. For SBS-modified asphalt, aging also causes the degradation of SBS, reducing its modification effectiveness.

Under the same aging time, the tensile strength of SBS-modified asphalt is consistently higher than that of 70# base asphalt. The existence of an SBS modifier increases the cohesiveness of the asphalt binder, allowing it to deform over a large range without breaking. Moreover, SBS-modified asphalt can effectively absorb and release stress, reducing stress concentration and the formation of micro-cracks. As a result, SBS-modified asphalt exhibits higher tensile strength. However, excessively high tensile strength is undesirable. For aging asphalt binders, an increase in tensile strength often indicates greater hardness and brittleness, which reduces its ability to deform.

**Figure 9:**
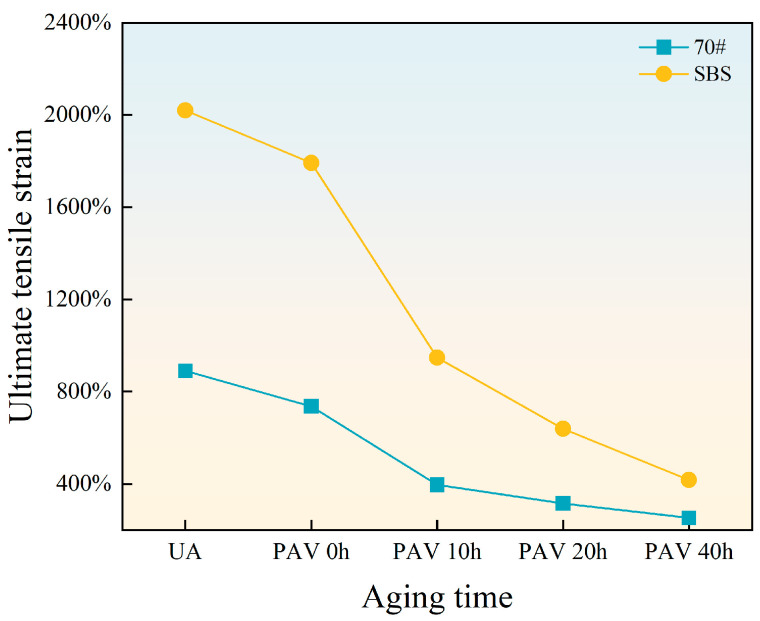
The ultimate tensile strain of asphalt binders.

Ductility refers to the total elastic and plastic deformation that asphalt binders can withstand under external force. Greater ductility represents smaller residual deformation of pavement under load and stronger resistance to cracking. In this study, the ultimate tensile strain is used as an indicator to evaluate the ductility of asphalt binders. Figure 9 presents the line diagram of the ultimate tensile strain of the two asphalt binders.

With increasing aging time, the ultimate tensile strain of both 70# base asphalt and SBS-modified asphalt decreases because aging weakens the ability of elongation. Compared with SBS-modified asphalt under the same aging time, the ultimate tensile strain curve of 70# base asphalt with aging time is gentler. The ultimate tensile strain of SBS-modified asphalt decreases significantly between short-term aging and 10 h long-term aging. The changing rate of SBS-modified asphalt is similar to that of 70# base asphalt between 20 h and 40 h long-term aging.

With increasing aging time, the ultimate tensile strain of both 70# base asphalt and SBS-modified asphalt decreases due to the reduced elongation capacity caused by aging. For 70# base asphalt, the ultimate tensile strain curve exhibits a gentler decline. Notably, the ultimate tensile strain of SBS-modified asphalt decreases significantly between short-term aging and 10 h of long-term aging. Between 20 and 40 h of long-term aging, the rate of change for SBS-modified asphalt becomes similar to that of 70# base asphalt.

The SBS modifier increases the ultimate tensile strain of asphalt binders, but aging leads to SBS degradation and destruction of the network structures, reducing its ability to resist cracking. From short-term aging to 10 h of long-term aging, SBS degradation has the most pronounced effect on ductility, causing a rapid reduction in ultimate tensile strain. As aging time increases, the degradation of SBS approaches saturation, reducing its impact on ductility. Consequently, the rate of decline in ultimate tensile strain slows with deeper aging levels.

### 3.2. Analysis of Frequency Sweep Test Results

#### 3.2.1. Stiffness Modulus and Creep Rate

The stiffness modulus (S) and creep rate (m) reflect the deformation and stress relaxation abilities of asphalt binders at low temperatures, respectively. Smaller S values and larger m values indicate better low-temperature performance. According to SHRP, at the design temperature, S should not exceed 300 MPa and m should not be less than 0.3 after 60 s of loading to meet the anti-cracking performance requirements. Figure 8 shows the evolution trends of S and m values with aging time at −6 °C, −12 °C, and −18 °C.

**Figure 10:**
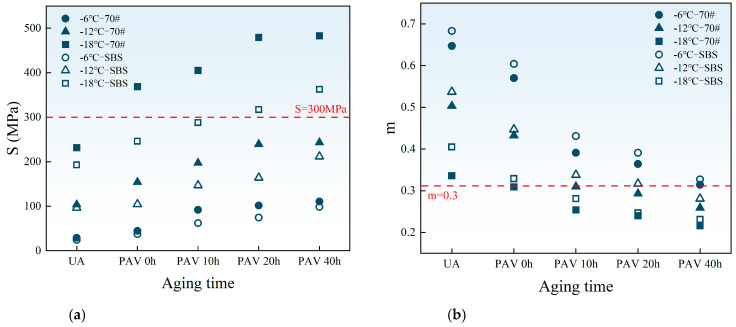
Values of S and m at different temperatures and aging levels: (**a**) values of S; (**b**) values of m.

As shown in Figure 10, with increasing aging time, the *S* value rises, while the *m* value decreases at the same temperature. This trend indicates that aging reduces the deformation capacity and stress relaxation ability of asphalt binders, thereby increasing the risk of cracking at low temperatures.

Additionally, lower temperatures further diminish the low-temperature cracking resistance of asphalt binders under the same aging levels. For the two asphalt binders, at the same aging time and temperature, SBS-modified asphalt exhibits a smaller *S* value and a larger *m* value compared to 70# base asphalt. This is because SBS improves the deformation ability of modified asphalt at low temperatures. It can absorb temperature stress more effectively and improve the stress relaxation performance of modified asphalt.

#### 3.2.2. G-R Parameter

As discussed in the Introduction Section, the traditional ductility test still has some limitations. Glover et al. [48] proposed the rheological parameter G′/(η′/G′), which established the relationship between asphalt pavement cracking and asphalt binder properties. This parameter showed a strong linear correlation with ductility within a certain range and could replace low-temperature ductility for evaluating asphalt binder cracking resistance. Rowe et al. [49,50] refined this parameter, introducing the Glover–Rowe (G-R) parameter to characterize asphalt binder cracking resistance. The G-R parameter exhibits a strong correlation with ductility and effectively characterizes the cracking tendency of asphalt binders [51,52]. This study adopted the G-R parameter as a substitute for the ductility indicator to evaluate the low-temperature cracking resistance of asphalt binders. When the G-R parameter exceeds 180 kPa, the asphalt pavement is at risk of cracking. If the G-R parameter exceeds 450 kPa, severe block and reflection cracks may develop. It is necessary to take measures to maintain and repair the road.

**Figure 11:**
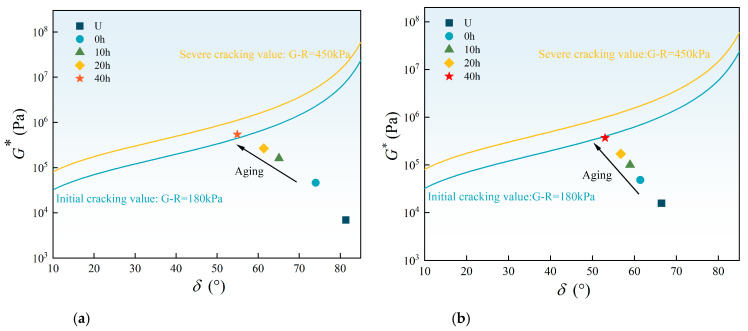
The black space diagrams of asphalt binders: (**a**) 70# base asphalt; (**b**) SBS-modified asphalt.

Figure 11 presents the black space diagrams of the two asphalt binders.

With increasing aging time, the data points shift from the lower right to the upper left in the diagrams, indicating a decrease in phase angle and an increase in complex modulus. After long-term aging of 40 h, the data point of 70# base asphalt has exceeded the initial cracking threshold of 180 kPa. Moreover, the G-R parameter of 70# base asphalt increases at a faster rate compared to SBS-modified asphalt. During the aging process, small and medium asphalt molecules polymerize into larger molecules through oxygen absorption, leading to an increase in modulus and a decrease in phase angle. Conversely, SBS decomposes into smaller molecules, disrupting the elastic network, which results in a decrease in modulus and an increase in phase angle. The higher cracking ratio of SBS after long-term aging mitigates the adverse effects of light component volatilization, oxidation, and hardening caused by thermal-oxidative aging. Consequently, the increase in the G-R parameter is moderated, reducing the cracking risk of SBS-modified asphalt at the same aging time.

**Figure 12:**
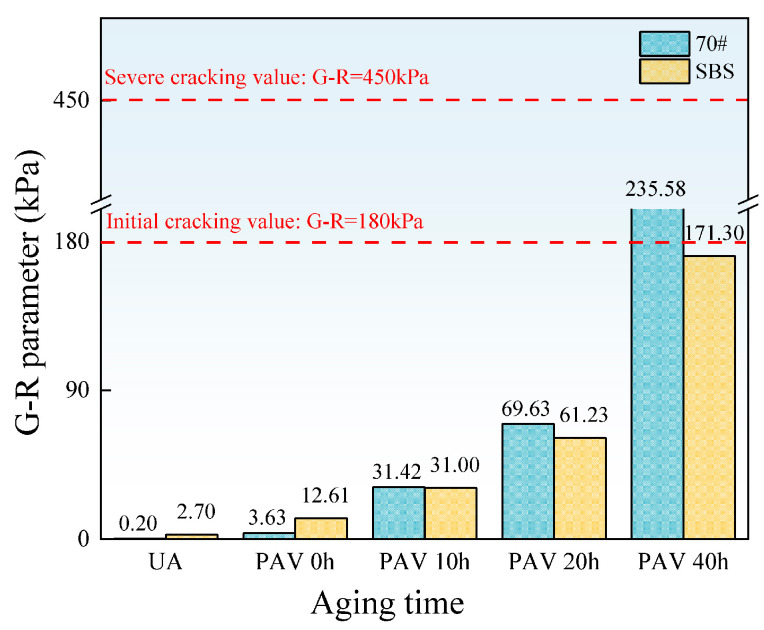
Values of the G-R parameter of asphalt binders.

Figure 12 shows the G-R parameter values of the two asphalt binders under different aging levels.

It can be seen from Figure 10 that the G-R parameter values of both asphalt binders increase with the aging time. In the non-aging and short-term aging stages, the G-R parameter of SBS-modified asphalt is higher than that of 70# base asphalt due to the higher elastic ratio provided by SBS. After long-term aging, the G-R parameter of SBS-modified asphalt becomes lower than that of 70# base asphalt and remains below the initial cracking threshold. This is because SBS adjusts the viscoelastic ratio of asphalt, thereby reducing the risk of cracking.

### 3.3. Correlation Analysis

Tensile strength and ultimate tensile strain measured by the poker chip test are fracture mechanics-based performance indicators that reflect the deformation characteristics of asphalt materials. In contrast, stiffness modulus, creep rate, and the G-R parameter characterize the viscoelastic and relaxation properties of asphalt. To verify the reliability of tensile strength and ultimate tensile strain in evaluating the low-temperature cracking resistance of asphalt binders, a correlation analysis with the other three parameters was conducted.

Pearson correlation analysis is a statistical method used to measure the linear relationship between two variables. Pearson correlation coefficient $r_{xy}$ is calculated to quantify the degree of correlation between the two variables, as shown in the Equation (10).

(10) $$r_{xy}=\frac{\sum x_{i}y_{i}-\bar{xy}}{(n-1)s_{x}s_{y}}=\frac{n\sum x_{i}y_{i}-\sum x_{i}\sum y_{i}}{\sqrt{n\sum x_{i}^{2}-{\left(\sum x_{i}\right)}^{2}}\sqrt{n\sum y_{i}^{2}-{\left(\sum y_{i}\right)}^{2}}}$$

where $r_{xy}$ is the Pearson correlation coefficient, $x_{i}$ is the value of each sample in the first variable, and $y_{i}$ is the value of each sample in the second variable.

The Pearson correlation coefficient ranges from −1 to 1. A negative value indicates a negative correlation between the two variables, while a positive value indicates a positive correlation. The larger the absolute value of the coefficient, the stronger the correlation; conversely, a smaller absolute value indicates a weaker correlation. Table 3 presents the calculated Pearson correlation coefficients.

It can be seen from Table 3 that the absolute values of the correlation coefficients between tensile strength, ultimate tensile strain, and stiffness modulus or creep rate exceed 0.9. The absolute values of the correlation coefficients between these two indicators and the G-R parameter exceed 0.7. This indicates that tensile strength and ultimate tensile strain are linearly correlated with stiffness modulus, creep rate, and the G-R parameter, with stronger correlations observed for stiffness modulus and creep rate.

Tensile strength and stiffness modulus both reflect the ability of asphalt to resist external forces, leading to a positive correlation. Likewise, ultimate tensile strain, which represents the maximum deformation capacity, is positively correlated with the creep rate, as the latter measures the rate at which asphalt releases stress through viscoelastic flow. However, the correlation between tensile strength, ultimate tensile strain, and the G-R parameter is weaker. The G-R parameter, a rheological indicator measured in the linear viscoelastic stage at a 1% strain level, characterizes the viscoelastic flow of asphalt under non-destructive conditions. Since the properties of asphalt in the damage stage differ from those in the linear viscoelastic stage, the G-R parameter is less effective in evaluating cracking resistance. In contrast, the poker chip test focuses on elastoplastic deformation and mechanical properties such as brittleness and fracture characteristics, resulting in weaker correlations with the G-R parameter.

In conclusion, the correlation analysis shows that the two parameters based on the poker chip test are effective indicators for evaluating the low-temperature performance of asphalt binders. Specifically, ultimate tensile strain reflects the maximum tensile deformation the asphalt binder film can achieve before fracture. This indicator effectively captures the ductility of asphalt binders, making it a valuable indicator for evaluating the cracking resistance of asphalt binders. Tensile strength and stiffness modulus can represent the stiffness of asphalt binders, while ultimate tensile strain and creep rate can represent the deformation ability of asphalt binders. Tensile strength obtained from the poker chip test, which is simple to prepare samples, fast to test, and can simulate the stress state of asphalt binder films, can be used as a substitute indicator of stiffness modulus. Similarly, ultimate tensile strain can be used as a substitute indicator of creep rate, especially in cases where DSR or BBR might not be a feasible option for research.

## 4. Conclusions

This study used the poker chip test to simulate the stress state of asphalt binders and analyze the tensile strength and ultimate tensile strain of 70# base asphalt and SBS-modified asphalt under various aging times. Frequency sweep tests and correlation analysis were conducted to verify the reliability of these indicators for evaluating low-temperature cracking resistance. The key findings are as follows:

1. With increasing aging time, the number of cavities on the failure cross sections of 70# base asphalt and SBS-modified asphalt increases, and the average cross-sectional area of cavities decreases. Under the same aging time, SBS-modified asphalt exhibits greater cohesiveness, a larger number of cavities, a smaller average cross-sectional area of cavities, and a flatter cross-sectional edge.
2. The stretching process can be divided into four stages. Tensile strength and ultimate tensile strain are sensitive to aging times and asphalt binder types. SBS-modified asphalt consistently shows higher tensile strength and ultimate tensile strain than 70# base asphalt. As aging time increases, tensile strength rises, while ultimate tensile strain decreases for both asphalt types.
3. According to the Pearson correlation analysis, tensile strength and ultimate tensile strain exhibit strong linear correlations with stiffness modulus and creep rate, with correlation coefficients exceeding 0.9. A linear correlation with G-R parameters was also observed, with correlation coefficients above 0.7. This indicates that tensile strength and ultimate tensile strain can serve as substitute indicators for stiffness modulus and creep rate, respectively.
4. Testing the tensile strength and ultimate tensile strain of asphalt binders can help in selecting the appropriate asphalt formulation to meet the low-temperature cracking resistance requirements, especially when DSR and BBR tests are difficult to perform.
5. In practical applications, especially in winter or cold regions, asphalt binders with higher ultimate tensile strain have greater potential in preventing low-temperature cracking of the pavement.

The tensile strength and ultimate tensile strain obtained from the poker chip test can evaluate the low-temperature cracking resistance of asphalt binders. Future studies should expand the test to include a wider range of asphalt types. Low-temperature cracking resistance evaluations using mixture specimens made from these asphalt binders should be performed to further validate the reliability of tensile strength and ultimate tensile strain as well.

## Figures and Tables

**Table 1:** Technical indicators of 70# base asphalt.

Technical Indicators	Units	Measured Data	Method
Penetration (25 °C, 100 g, 5 s)	0.1 mm	72	ASTM D5
Ductility at 15 °C	cm	>100	ASTM D113
Softening Point	°C	47	ASTM D36
Flash Point	°C	282	ASTM D92
Density at 25 °C	g/cm^3^	1.030	ASTM D70

**Table 2:** Technical indicators of SBS-modified asphalt.

Technical Indicators	Units	Measured Data	Method
Penetration (25 °C, 100 g, 5 s)	0.1 mm	49	ASTM D5
Ductility (5 °C, 5 cm/min)	cm	31	ASTM D113
Softening Point	°C	78.5	ASTM D36
Flash Point	°C	295	ASTM D92
Relative Density at 25 °C	—	1.033	ASTM D70

**Table 3:** The calculated results of Pearson correlation coefficients.

$r_{xy}$	−6 °C *S*	−6 °C *m*	12 °C *S*	12 °C *m*	18 °C *S*	18 °C *m*	G-R Parameter
70# T	0.975	0.979	0.958	0.972	0.911	0.992	0.849
70# $\sigma_{m}$	0.999	0.999	0.981	0.998	0.941	0.994	0.705
SBS T	0.995	0.960	0.997	0.935	0.979	0.921	0.930
SBS $\sigma_{m}$	0.975	0.995	0.957	0.978	0.968	0.961	0.792

## Data Availability

The data presented in this study are conditionally available upon request from the corresponding author.
